# Three Cases Illustrating the Necessity of Vagical Examination in Obscure Cases of Reflex Pain

**Published:** 1890-03

**Authors:** G. H. Noble

**Affiliations:** Atlanta, Ga.


### THREE CASES ILLUSTRATING THE NECESSITY OF VAGICAL EXAMINATION IN OBSCURE CASES OF REFLEX PAIN.

BY G. H. NOBLE, M. D., ATLANTA, GA.

We not unfrequently meet with obscure cases pronounced incurable, which, when traced to the true cause, proves simple in the extreme. They are examples of the violation of one of the first principles of diagnosis; that is, the examination of every organ in the body.

As an illustration, I shall select three out of quite a number of cases I have met with.

The first was a resident of this city, a lady of billious temperament, and mother of eight children. She enjoyed good health all of her life except an intollerable itching of the parts supplied by the external nasal branch of the opthalmic nerve. The paroxysms would come several times a day and last for more than an hour each time. So distressing were the attacks that she was in fear of losing her mind. She had exhausted the skill of many reputable physicians and neurologists, north and east, without any relief, and had received nearly as many explanations of the causes as she had physicians, except, perhaps, indigestion and catarrh of the bile dutcs were in the head.

She was questioned closely and examined carefully except for diseases of the pelvic organs, she having stated "that she felt no inconvenience and had no symptoms of uterine disease."

But afterward the fact was elicited that she had metrororrhagia, which she attributed to the change of life and regarded as normal flushings. We discovered a bilateral laceration of the cervix extending into the vagnial junction, subinvolution with considerable ingorgement, and some other minor symptoms usually attending such conditions. It was then suggested to her that the itching might be a reflex disturbance, dependent upon the condition of the uterus, and advised treatment and repair of the cervix, to which advice she readily assented.

Under treatment by the glycerine tampon the paroxysms grew less severe and frequent, until the day the laceration was closed, when they disappeared entirely.

She has now passed a period of eight years, without the slightest evidence of return of the disease.

The second case was a large, fleshy lady, from Chillocootia, Mo. She was anæmic and slightly chloratic. She suffered from an intollerable dyspepsia, for which she had received no relief from the many professional men she had consulted.

She gave no history of uterine diseases, but as a last resort an examination was made with the hope of finding some explanation of the matter. We found about the same condition as in the above case, and gave the same advice. She experienced relief at times during the treatment, but was not materially benefitted untl the cervix was closed, then like magic the dyspepsia disappeared, and for two years remained well. Since that time I have heard nothing from her, and am unable to relate her condition.

The third case was a lady from Indiana. Aet. 35. Married 13 years. Had three children. Her bad health began six months after last confinement, four years ago. She became very weak, but afterwards recovered and moved to Montgomery, Ala., where she had some malarial impressions, but again recovered, and returned to her home in Indiana, but continued to have pains at the lower angle of the scapula, and intercostal neuralgia of the left side, and many pains from the bowels (abdomen) to the limbs. When located in the bowels she claimed that it caused dysentary. She had no pain with menstration, nor other menstrual troubles. She was sometimes constipated, (hard, lumpy actions) generally alternating with diorrhœa, which would last for days, and even months. Had heavy, weighty sensation in the stomach, especially after eating. A change in diet seemed to have no effect upon the symptoms. Head felt heavy and stupid, and claimed to have high fever at times. Pains were worse in the daytime and lost flesh and strength steadily. The pains in the shoulders and intercostal neuralgia were constant.

The above history naturally directed my attention to constipation, and I expressed my opinion that it was the probable cause of the trouble. She stated, however, that the same opinion had been advanced before, and that attention to the constipation did not relieve her of the pains. However, I gave her a prescription for the condition of the bowels ; also one of aconita for the neuralgia. I promised her nothing, as she refused a vaginal examination, and I could not attribute the symptoms to disorder of any other organs. She returned in two weeks, unimproved, and complained of a slight leucorrhœa. I made a vaginal examination, and found a laceration of the cervix, extending externally to the vaginal junction, and internally a white cicatritial line reaching to the internal os, also subinvolution, ingorgement, endometrites and erosion of the os.

I stopped all medication exceptthe laxative, and resorted to the glycerine tampon, which offered her slight relief.

The day the cervix was thoroughly dilated and the old scar softened and stretched, the pain disappeared.

At the end of a month I left off the cervical tampon, and the pain returned. It was again resumed, and the pains disappeared.

These cases fully illustrate the necessity of vaginal examinations in obscure cases when the cause of pain cannot be ascribed to disease in other parts of the body.

Especially are we liable to be thrown off our guard in making our diagnosis where there are no symptoms of disease of the uterus and appendage.

